# A Practical Treatise on Surgical Diagnosis

**Published:** 1884-07

**Authors:** 


**A Practical Treatise on Surgical Diagnosis.** Designed as a Manual for Practitioners and Students of Medicine. By AMBROSE L. RANNEY, M. D., Professor of Practical Anatomy in the New York Post Graduate Medical School, etc. Third edition, thoroughly revised, enlarged and properly illustrated. New York: Wm. Wood & Co. 1884.

The first edition of this work appeared in 1879. That it already has reached a third edition is conclusive evidence of its value. Both the author and the publishers seem determined that it shall continue to deserve professional favor, for the present edition of the work is materially improved by the addition of new chapters upon diseases of the spinal cord and their envelopes, a careful revision of the whole work and the introduction of many excellent illustrations. The plan of the work is, we think, an excellent one, in that for the student the presentation of the symptoms of diseases in marked contrast is an aid to memory, and to the practicing physician it is a convenient reference on questions of diagnosis. We know of no better book of the kind, and its popularity with the profession will undoubtedly continue and increase. The publishers have issued it in excellent form.

